# Immune System Alterations in the Development of Three Urological Cancers: Insights from Large-Sample Mendelian Randomization

**DOI:** 10.3390/biomedicines13061480

**Published:** 2025-06-16

**Authors:** Zhijian Chen, Ye Xie, Xiong Chen, Guibin Hong, Runnan Shen, Haishan Lin, Fan Jiang, Yun Wang, Mengyi Zhu, Yixuan Liu, Haoxuan Wang, Hongkun Yang, Tianxin Lin, Shaoxu Wu

**Affiliations:** 1 Department of Urology, Sun Yat-sen Memorial Hospital, Sun Yat-sen University, Guangzhou 510120, China; chenzhj87@mail2.sysu.edu.cn (Z.C.); xiey235@mail2.sysu.edu.cn (Y.X.); chenx955@mail.sysu.edu.cn (X.C.); honggb3@mail2.sysu.edu.cn (G.H.); shenrn3@mail2.sysu.edu.cn (R.S.); linhsh23@mail.sysu.edu.cn (H.L.); jiangf55@mail2.sysu.edu.cn (F.J.); wangy676@mail2.sysu.edu.cn (Y.W.); zhumy25@mail2.sysu.edu.cn (M.Z.); wanghx63@mail2.sysu.edu.cn (H.W.); yanghk3@mail2.sysu.edu.cn (H.Y.); 2 Sun Yat-sen University Cancer Center, Sun Yat-sen University, Guangzhou 510060, China; liuyx4@sysucc.org.cn; 3 Guangdong Provincial Key Laboratory of Malignant Tumour Epigenetics and Gene Regulation, Sun Yat-sen Memorial Hospital, Sun Yat-sen University, Guangzhou 510000, China; 4 Guangdong Provincial Clinical Research Centre for Urological Diseases, Guangzhou 510000, China

**Keywords:** urological cancer, bladder cancer, prostate cancer, kidney cancer, Mendelian randomization, immunity

## Abstract

**Background:** Urological cancers (UCs) greatly impact global public health. While immunity plays an important role, the contribution of specific immune cell traits to the development of UCs remains unclear. In our study, we employed Mendelian randomization (MR) to elucidate the causal relationship between 731 immune cell traits and three common UCs, namely kidney cancer (KC), bladder cancer (BC), and prostate cancer (PC). **Methods:** In our research, we adopted and preprocessed the statistics of 731 immune cell types from the GWAS Catalog. The data of three common UCs were acquired from two databases, FinnGen and IEU. Five MR analysis models, including random-effect inverse-variance weighted, weighted median, MR Egger, weighted mode, and simple mode, were used to assess the association between 731 immune cell traits and UCs. Subsequently, a meta-analysis of the IVW method was performed, and the significant results were analyzed using the reverse MR method. Sensitivity analyses, including leave-one-out analysis, were also performed. **Results:** When analyzing the two datasets separately, 25, 41, and 23 immune phenotypes were found to be significantly associated with BC, PC, and KC, respectively. When applying meta-analysis, the combined results showed that a total of 18 immune cell types manifested the significant association, including 4 and 14 immune cell traits regarding BC and PC, respectively. Utilizing reverse MR analysis on the combined results, we found that two immune cell traits, namely lymphocyte absolute cell counts and CX3CR1 on CD14+ CD16- monocytes, showed a reverse causal relationship with PC. **Conclusions:** Our research depicts the immune landscape for these three common UCs, highlighting their strong genetic associations with immune cells. It provides valuable insights for identifying the systemic immunological context of cancer susceptibility and the development of blood-based immunological biomarkers and therapeutic targets.

## 1. Introduction

Urological cancers (UCs) account for nearly 13% of all cancers, substantially impacting global public health, especially in an aging society [1,2,3]. UC encompasses three principal malignancies: prostate cancer (PC), bladder cancer (BC), and kidney cancer (KC). According to reports, PC is the second leading malignancy in men, with an estimated 1.4 million newly diagnosed cases each year [4]. BC and KC ranked as the 10th and 14th most common cancers, respectively, with roughly 573,000 and 430,000 new cases annually, according to the estimated data [5].

Immune system disorders affect the development of UCs, while the immune system of patients with UC also changes, influencing the progression and prognosis of cancer. Researchers have highlighted the complex relationship between UCs and the immune system, mainly focusing on the alteration of the immune system in patients with UCs. In muscle-invasive BC (MIBC), the quantification of tumor-infiltrating lymphocytes located in the tumor-adjacent stroma was reported to predict immune phenotype, molecular subtype, and patient outcome [6]. In non-muscle-invasive BC (NMIBC), studies suggested that PDL1+ Treg cells were induced after Bacillus Calmette–Guerin instillation [7]. For PC, it was reported that the initiation, progression, and development of castration-resistance involved the interaction of the host immune system and tumor cells [8]. Specifically, neuroendocrine differentiation (NED), induced by sustained androgen receptor inhibition, was facilitated by immune cells, resulting in the evasiveness of tumor cells under drug exposure [9,10]. For KC, research revealed that T cells made up the predominant type of immune cell in the microenvironment of clear-cell renal-cell carcinoma (ccRCC), accounting for an average of 51% [11]. The infiltrating T cells displayed an immunosuppressed phenotype, which partially explains the continued progression of ccRCC despite substantial T cell infiltration [12]. However, studies investigating the relationship between immune imbalance and tumor development remain limited, and conventional studies may introduce bias owing to selection bias, reverse causation, and the influence of multiple confounding factors.

Mendelian randomization (MR) utilizes genetic variants (single-nucleotide polymorphisms, SNPs) as instrumental variables (IVs) of the exposure, which examine the cause-and-effect relationship with the outcome while reducing bias introduced by confounding factors; it is being increasingly used for inferring the causal relationships between traits. Several studies have adopted MR to infer the causality between immune cell traits and cancers, including breast cancer and malignant neoplasm of the ovary [13,14]. The aim of this study was to investigate the potential causal relationships between 3 common urological cancers and 731 immune cell traits using MR analysis. The results of our study can contribute to the development of blood-based biomarkers for early risk assessment, guide patient stratification, and inform future directions in immunotherapy research for kidney, bladder, and prostate cancers.

## 2. Method

### 2.1. Study Design

**Figure 1:**
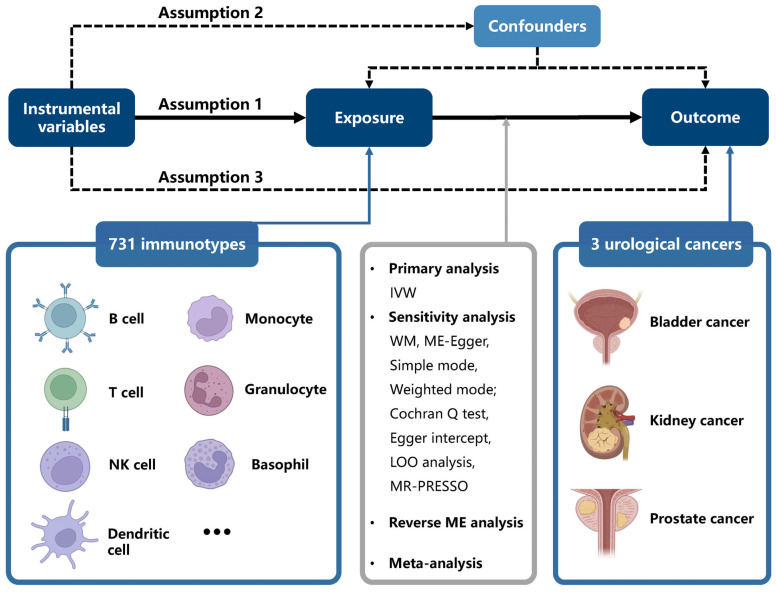
Study design of Mendelian randomization analysis of immune cell signatures in UCs. All figures were produced with BioRender (https://app.biorender.com/)(accessed on 16 February 2025).

By using SNPs, which are randomly allocated in offspring, as IVs, instrumental-variable analysis mimics a randomized controlled trial (RCT) to examine causal association while remaining unaffected by confounding factors such as age and region. Additionally, this MR analysis is based on three assumptions: [1] SNPs are strongly associated with exposure; [2] SNPs are not associated with any confounders of the exposure–outcome relationship; [3] SNPs influence the outcome exclusively through exposure. In this study, we used public datasets to infer causality, and no additional ethical approval was needed owing to prior received approval. A brief overview of the study is displayed in Figure 1, and our research adhered to STROBE-MR guidelines (Supplementary Table S24).

### 2.2. GWAS Data Sources

The immune cell data were acquired from the publicly accessible GWAS Catalog (https://www.ebi.ac.uk/gwas/ (accessed on 16 February 2025)) [15,16], covering registration codes from GCST90001391 to GCST90002121, which were originally derived from Dr. Valeria Orrù et al. [17]. The study used 731 immune cell traits, including 389 median fluorescence intensities (MFIs), which reflected surface antigen levels, 32 morphological parameters (MPs), 118 absolute cell counts (AC), and 192 relative cell counts (RCs). In detail, MFI, AC, and RC features encompassed immune cell panels including TBNK (T cells, B cells, natural killer cells), mature-stage T cells, Treg cells, B cells, myeloid cells, monocytes, and cDCs, while MP features mainly included TBNK and cDCs.

The genetic data regarding the three common UCs—BC, PC, and KC—were sourced from two databases, including the FinnGen database and the Open GWAS database. Informed consent was acquired from European individuals included in the study cohort. The GWAS IDs for data from the Open GWAS database were ieu-b-4874, ieu-b-85, and ukb-b-1316 for BC, PC, and KC, respectively. More details can be found in the Supplementary Materials. Notably, there was no overlap between the two cohorts mentioned above, indicating the feasibility of implementing a two-sample MR study.

### 2.3. Selection Criteria for Instrumental Variables

To satisfy the relevance assumption between SNPs and the exposure, a significance threshold of 1 × 10^−5^ was applied. Additionally, SNPs with strong linkage disequilibrium were then filtered (r^2^ < 0.001), at a window size of 10,000 kb. In addition, to address potential estimation bias generated by weak IVs, we estimated the F-statistic. Weak IVs with F-statistics < 10 were subsequently eliminated. Moreover, the minor allele frequency was used to prune exposures related to rare variants, with a threshold of 0.01. Lastly, palindromic SNPs were also removed, and filtered SNPs were harmonized to guarantee consistency in the estimation of causal effects for the same effect allele.

### 2.4. Mendelian Randomization Analysis

Five MR methods, namely random-effect inverse-variance weighted (IVW), MR Egger, weighted median, weighted mode, and simple mode, were used to assess the association between exposure and outcome. The IVW method was used as the primary approach to estimate the causal effects. For each IV, the effect size was estimated using the Wald ratio (WR). Weighted median (WM) and MR Egger were utilized to generate unbiased estimates with invalid IVs, which can improve the robustness of IVW estimates, in spite of their inefficiency (wider CIs). MR Egger allows for pleiotropic effects that are uncorrelated with the variant–exposure relationships, while WM assumes that over 50% of the IVs are valid to yield unbiased estimates. To guarantee the robustness of our findings, the leave-one-out analysis was performed.

To evaluate pleiotropy, MR Egger intercept analysis was performed. Heterogeneity was tested using Cochran’s Q statistic, and I^2^ statistics were computed for evaluation. Heterogeneity was considered absent (I^2^ < 25%) or mild (I^2^ < 50%), according to I^2^ statistics. To enhance the robustness of our results, we selected the significant results from one database and conducted a meta-analysis of the IVW-MR estimates across the two data sources. Moreover, reverse MR analysis was conducted to infer bidirectional causality regarding the significant immune traits identified by meta-analysis.

### 2.5. Evaluation of Ancestry-Specific Causal Associations

To explore potential differences in causal relationships across ancestries, we performed an MR analysis using data from a non-European population. Specifically, we searched the IEU Open GWAS catalog and only identified a PC GWAS conducted in the Biobank Japan (BBJ) cohort (GWAS ID: bbj-a-148). This dataset included 109,347 participants, among whom 5408 were diagnosed with PC. A *p*-value of less than 0.05 was considered statistically significant.

### 2.6. Statistical Analysis

Statistical analyses were conducted using R version 4.2.1 (The R Project for Statistical Computing; https://www.r-project.org/ (accessed on 16 February 2025)). “ldscr” and “TwoSampleMR” packages were used. To control for multiple comparisons, Bonferroni correction was applied based on the number of primary hypotheses tested. Specifically, our study investigated the potential causal associations between 3 distinct urological cancers and 731 immune cell traits using MR analysis. Since the primary interest was the three cancer types—each considered an independent outcome—the significance threshold was adjusted by dividing 0.05 by 3 (i.e., *p* < 0.0167) to account for testing across the three cancer types [13]. When evaluating the meta-analysis, reverse MR analysis, heterogeneity, and horizontal pleiotropy, a *p*-value of less than 0.05 was considered statistically significant.

## 3. Results

### 3.1. Causal Relationship Between Immune Cell Traits and BC

**Figure 2:**
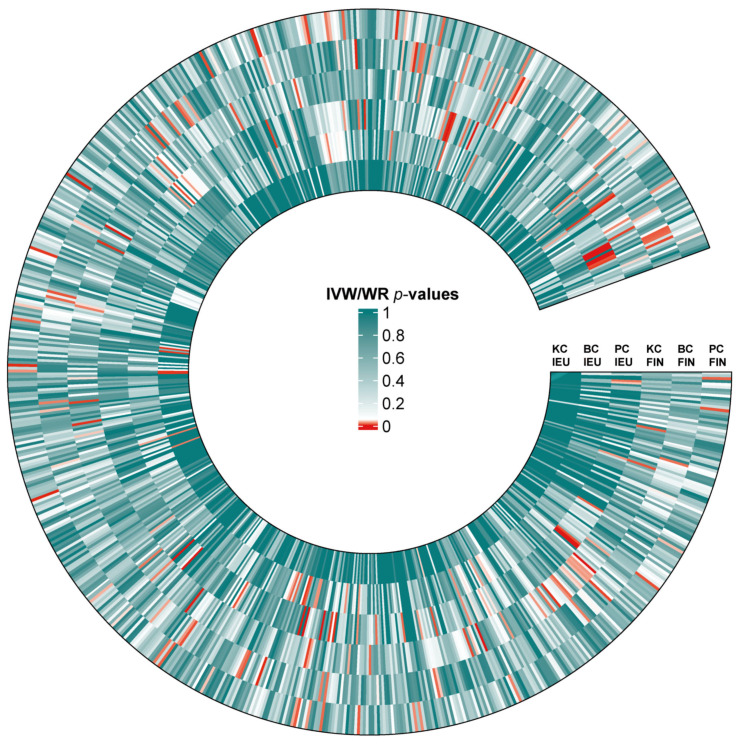
Heat map of IVW/WR estimates for three urological cancers from two databases. Standardized beta values of the inverse-variance weighted or Wald ratio (IVW/WR) are shown for associations between blood immune traits and kidney cancer (KC), bladder cancer (BC), and prostate cancer (PC) across two GWAS sources: IEU and FinnGen (FIN). The color of each cell represents the *p*-value of IVW/WR methods.

**Figure 3:**
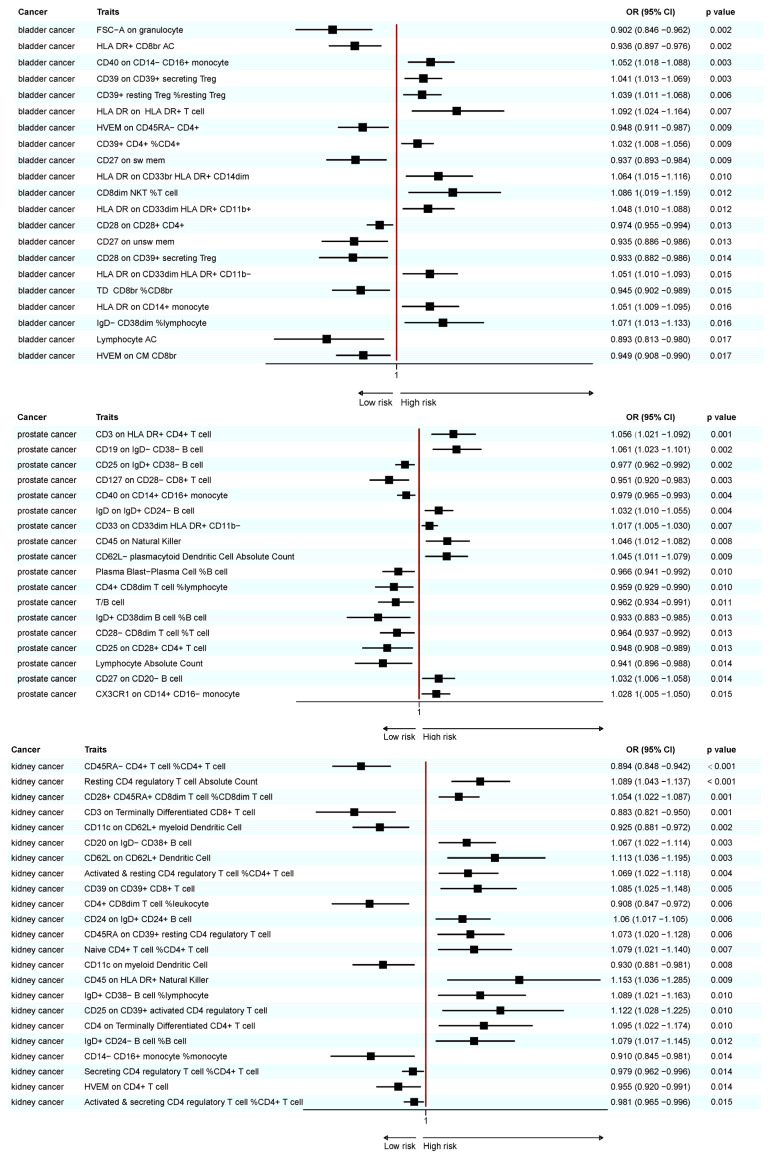
Forest plot of significant MR results in the FinnGen database.

**Figure 4:**
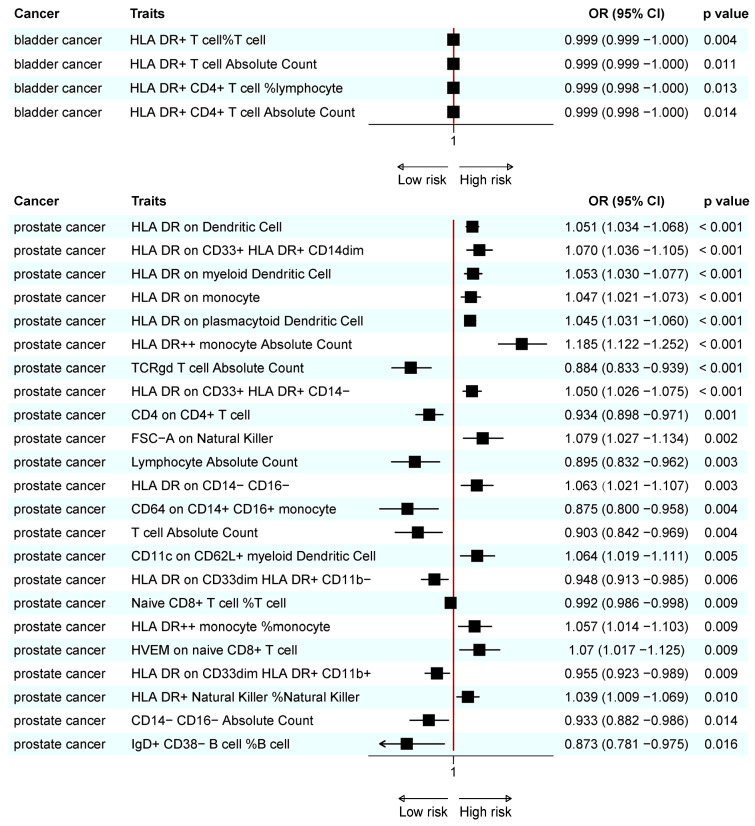
Forest plot of significant MR results in the IEU database.

A total of 25 immunotypes were significantly associated with BC based on the IVW method. Specifically, two immune cell traits from the TBNK panel, three from the Treg panel, one from the B cell panel, three from the myeloid panel, and two from the monocyte panel showed a positive association with BC risk. In contrast, six immune cell traits from the TBNK panel, three from the T cell maturation stages panel, two from the Treg panel, two from the B cell panel, and one from the cDCs panel demonstrated a negative association (Figure 2, Figure 3 and Figure 4; Supplementary Tables S3 and S13).

In general, only three immunotypes, including HLA DR on CD33br HLA DR+ CD14dim, HLA DR on CD33dim HLA DR+ CD11b+, and HLA DR on CD14+ monocytes, showed mild heterogeneity, and none of the immune traits showed pleiotropy (Table 1; Supplementary Tables S4, S5, S13 and S14).

### 3.2. Causal Relationships Between Immune Cell Traits and PC

A total of 41 immune cell traits were significantly associated with PC. Specifically, immune cell traits of TBNK (n = 6) and the maturation stages of T cells (n = 1), B cells (n = 3), myeloid cells (n = 3), monocytes (n = 3), and cDCs (n = 5) were positively associated with PC risk. Conversely, immune cell traits of TBNK (n = 6) and the maturation stage of T cells (n = 2), Tregs (n = 3), B cells (n = 4), myeloid cells (n = 2), and monocytes (n = 3) showed a negative association (Figure 2, Figure 3 and Figure 4; Supplementary Tables S6 and S15).

Generally, for PC, four immunotypes exhibited heterogeneity, including CD3 on HLA DR+ CD4+ T cells, the proportion of CD4+ CD8dim T cells within lymphocytes, HLA DR on CD14- CD16-, and HLA DR on CD33dim HLA DR+ CD11b-. The proportion of CD4+ CD8dim T cells within lymphocytes and HLA DR on myeloid dendritic cells were the only two immune traits that demonstrated pleiotropy (Table 1; Supplementary Tables S7, S8, S16 and S17).

### 3.3. Causal Relationship Between Immune Cell Traits and KC

A total of 23 immune cell traits were significantly associated with KC (Figure 2, Figure 3 and Figure 4; Supplementary Tables S9 and S18). To elaborate, one TBNK trait, two maturation stages of T cell traits, six Treg traits, four B cell traits, and one cDC trait demonstrated a positive association with an elevated risk of KC. On the contrary, one TBNK trait, three maturation stages of T cell traits, two Treg traits, one monocyte trait, and two cDC traits were negatively associated with KC risk.

In total, as for KC, only one immunotype named CD11c on myeloid dendritic cells showed mild heterogeneity. CD39 on CD39+ CD8+ T cells and CD24 on IgD+ CD24+ B cells were the only two immunotypes that exhibited pleiotropy (Table 1; Supplementary Tables S10, S11, S19 and S20).

### 3.4. Combined Results for Urological Cancers from Meta-Analysis and Reverse MR Analysis

**Figure 5:**
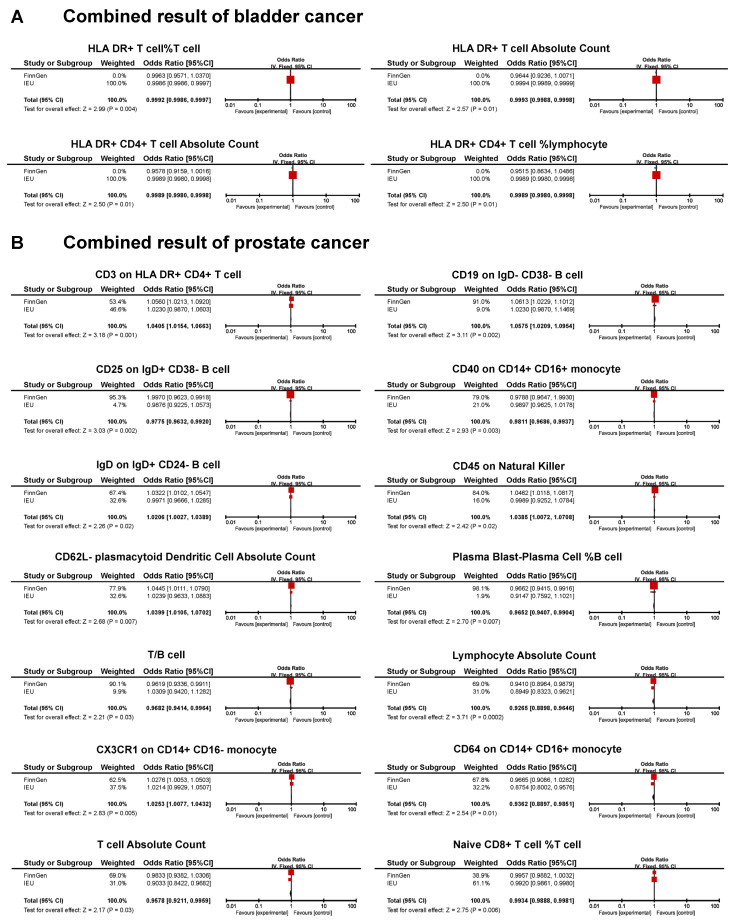
Forest plot of the significant combined result of MR analysis. (**A**) Significant combined result of BC; (**B**) significant combined result of PC.

A meta-analysis found that 18 immunotypes exhibited a significant association with UCs (Figure 5).

The combined results confirm the negative causal risk of the proportion of HLA DR+ T cells within T cells, HLA DR+ T cell AC, HLA DR+ CD4+ T cell AC, and the proportion of HLA DR+ CD4+ T cells within lymphocytes in BC. In PC, the results of the meta-analysis confirmed the positive relationship of CD3 in HLA DR+ CD4+ T cells, CD19 on IgD- CD38- B cells, IgD on IgD+ CD24- B cells, CD45 on natural killer cells, CD62L- plasmacytoid dendritic cell AC, and CX3CR1 in CD14+ CD16- monocytes. In addition, a negative association was found for CD25 on IgD+ CD38- B cells, CD40 on CD14+ CD16+ monocytes, the proportion of plasma blast-plasma cells within B cells, T/B cells, lymphocyte AC, CD64 on CD14+ CD16+ monocytes, T cell AC, and the proportion of naive CD8+ T cells within T cells. In KC, none of the immunophenotypes was found to be significantly associated with the risk (Figure 5).

Reverse MR analysis was applied to the significant results obtained from meta-analysis. We found that PC was associated with lower levels of lymphocyte AC and CX3CR1 on the CD14+ CD16- monocyte. The remaining sixteen immunotypes did not show significant results (Table 2; Supplementary Table S21).

### 3.5. Differences in Causal Relationship Across Ethnic Groups

In total, 30 immune cell traits were found to be causally associated with the risk of PC in the BBJ dataset. Among these, 12 immune cell traits showed positive association, while 18 immune cell traits manifested the opposite results (Supplementary Table S25). Notably, none of these associations were directionally consistent with those identified in the meta-analysis of the European cohort.

## 4. Discussion

Harnessing a wide range of GWAS data, we investigated the causal connections between 731 immune cell traits and 3 common UCs: BC, PC, and KC. Our study employed meta-analysis to guarantee the robustness of the results. When applying only MR analysis, we identified 89 immune cell traits associated with UCs. As for meta-analysis, 18 immune cell traits only in BC and PC were identified as causally associated, 2 of which were found to be significantly associated in the reverse MR analysis.

BC can be mainly divided into two categories according to its T stage, which are MIBC and NMIBC. MIBC accounts for about 50–60% of overall cases and is the primary cause of death in BC patients owing to its invasiveness. For MIBC patients at cT2-4aN0M0, cisplatin-based combinatory neoadjuvant chemotherapy is advocated. In recent years, with research investigating the effectiveness of anti-PD-1/PD-L1 drugs, chemoimmunotherapy has aroused the public’s attention for its capability of improving patients’ survival. Regarding adjuvant therapy, immunotherapy also served as a possible option. For patients at an elevated risk of recurrence following surgery, the FDA approved the administration of nivolumab, which is a classic agent targeting PD-1. Therefore, it is important to describe the immune landscape of BC for the purpose of more efficient utilization of immunotherapy [18].

Previously, Li et al. reported 16 immune cell traits associated with BC using the MR method [19]. However, in our study, only four immune cell signatures were identified as causally associated through meta-analysis, all of which showed negative association with the risk of BC. HLA DR+ CD4+ T cell was identified as a vital cell type decreasing the risk of BC. HLA DR belongs to class II major histocompatibility complex molecules in the human leukocyte antigen system, which are predominantly expressed on certain immune cells’ surfaces, such as antigen-presenting cells, including dendritic cells, B cells, and macrophages. During immune responses, antigen-presenting cells express the complex of HLA-DR molecules and exogenous or endogenous antigenic peptide fragments and interact with the T cell receptors on CD4+ T cells, thereby activating the T cells. When expressed on T cells, they were identified as a late-stage activation marker [20]. It is acknowledged that CD4+ T cells could differentiate into immune-stimulating and immunosuppressive cells, the balance of which plays a pivotal role in the tumor environment [21]. Drawing from our result, HLA DR+ CD4+ T cells, whose immune-activated subtype outnumbered the immunosuppressive subtype, were assumed to show a pivotally protective role against the onset of BC. Furthermore, HLA DR+ CD4+ T cells were significantly enriched in the peripheral blood of patients responding to immunotherapy in a pan-cancer study, demonstrating their potential for predicting immunotherapy responses [22]. However, another study showed the opposite result when analyzing patients with lung cancer, which may be explained by the heterogeneous function of HLA DR+ CD4+ T cells in different cancers. Future studies are needed to explicitly explain the crucial effect of HLA DR+ CD4+ T cells on BC.

For PC, surgery or radiation therapy is recommended for localized disease, although the recurrence rate is still difficult to control (25–35%) [23]. Regarding recurrent PC, androgen deprivation therapy targeting androgen receptors is considered the standard treatment. Yet, by inducing microenvironment changes, like NED, prostate tumor cells could adapt to androgen deprivation and evolve into a more aggressive form, which is known as castration-resistant PC. In recent years, immunotherapy has served as an alternative treatment for patients. However, since PC is a frustrating, immunogenic “cold” tumor, characterized by an immunosuppressive microenvironment like restricted CD8+ T cells, patients receive unsatisfactory results when treated with immunotherapy [24]. Hence, it is crucial to investigate the links between the immune system and PC, providing insights into potential predictors or targets for new agents.

In PC, a meta-analysis identified 14 immune cell signatures linked to risk, comprising 6 risk factors and 8 protective factors. In the former study, authors found that three immune cell traits increased the risk of PC [25]. However, in our meta-analysis, none of these results showed significance, which may be explained by the bias introduced by drawing from only one database [25]. Notably, two immune cell traits, named lymphocyte absolute count and CX3CR1 on the CD14+ CD16- monocyte, were found to be significant in reverse MR analysis. Regarding the former, lower levels were associated with a higher risk of PC, and PC, in turn, diminished the count of lymphocytes, which contributed to an unceasingly decreased level. The bidirectional relationship in the case of the latter was different. Higher levels of CX3CR1 on CD14+ CD16- monocytes elevated the risk of PC, while PC downregulated its level as a negative feedback mechanism. CX3CR1 is a chemokine receptor majorly involved in regulating monocyte chemotaxis and tumor angiogenesis. It was speculated in another Mendelian randomization study that CX3CR1 on CD14+ CD16- monocytes may promote the onset of PC through the CX3CR1/CX3CL1 signaling pathway, which is actively involved in promoting tumor angiogenesis, migration, and infiltration [25]. When PC develops, we assume that CX3CR1 may be downregulated, which is consistent with a prior study measuring the level of CX3CR1 expression in PC in contrast with adjacent normal tissue [26].

In KC, immunotherapy was recommended as the first-line standard option for patients with intermediate/poor-risk metastatic tumors. In spite of these important advancements, only minor patient cohorts achieved longstanding disease control in RCC populations. Hence, elucidating the relationship between the immune system and KC is of high significance. In KC, 23 immune cell signatures were regarded as significant factors, including 13 risk factors and 10 protective factors, through analyzing FinnGen data. However, results from the IEU dataset analyzed by IVW analysis retrieved no significant results, which was also consistent with the inefficiency of immune therapy for KC to a certain extent. Since meta-analysis was not applicable, the significant results obtained only from the FinnGen dataset, with relatively lower robustness, should be interpreted with caution.

It is important to note that the immune cell traits examined in this study were measured in peripheral blood. While these systemic immune traits may not fully represent the complex and spatially organized immune landscape within the tumor microenvironment, they nonetheless provide valuable insights into general immune status and regulation across individuals. Blood-based immune markers are widely used in clinical and epidemiological research due to their accessibility, reproducibility, and compatibility with large-scale genomic datasets. Moreover, accumulating evidence suggests that systemic immune dysregulation can influence tumorigenesis, modulate host immune surveillance, and affect responses to therapy. Therefore, although our findings should be interpreted with caution regarding direct relevance to tumor-infiltrating immune cells, they offer meaningful information about the systemic immunological context of cancer susceptibility and may contribute to the development of blood-based immunological biomarkers and therapeutic targets for future translational studies.

As acknowledged, immune phenotypes can be shaped by a wide range of factors, including genetic background, environmental exposures, lifestyle habits, and even psychological status [27]. These factors vary substantially across ethnic groups, particularly with respect to immunogenetic architecture [28]. For example, a comprehensive pan-ancestry analysis by Dr. Jian Carrot-Zhang and colleagues involving 10,638 patients from The Cancer Genome Atlas (TCGA) revealed significant ethnic differences in genomic and immunologic profiles. Notably, increased FBXW7 mutations were observed in patients of African descent, while VHL and PBRM1 mutations were decreased in African-origin renal cancer patients. Additionally, East Asian patients with bladder cancer exhibited reduced immune activity compared to European patients [29]. These findings underscore the biological rationale for caution when extrapolating MR results across ancestries. Therefore, MR associations derived from European populations may not be directly applicable to non-European groups. However, large sample GWAS datasets for UCs in non-European ancestries remain limited, with PC in East Asians being a notable exception. To explore it further, we conducted replication MR analysis using Japanese GWAS data to reveal the causal effect between 731 immune cell traits and PC risk. We found that 30 immune cell traits were causally associated with the risk of PC, while none of these associations were directionally consistent with those observed in meta-analyses in European-ancestry cohorts. Further studies are warranted to investigate immune cell traits associated with other cancer types in diverse ancestral populations.

There exist several limitations in our research. Firstly, MR analysis simply provides evidence for unidirectional causality linking immune cell traits to three common UCs; thus, comprehensive molecular experiments are needed to discover the actual mechanisms related to specific immune cell traits and cancer. Additionally, it is widely recognized that interactions between cells in the tumor microenvironment are common, which implies that analyzing the effect of a single immune cell trait on cancer could introduce bias. Lastly, heterogeneity and pleiotropy were found in some immune cell traits when analyzing only one dataset, which could have distorted the findings. Fortunately, only mild heterogeneity existed for the significant results obtained by meta-analysis.

## 5. Conclusions

In our study, we uncovered the immune landscape for three common UCs—BC, PC, and KC—by analyzing two datasets separately. To emphasize some specific immune traits, we adopted a meta-analysis strategy, demonstrating causal links between 18 immune cell traits and cancers with robustness. Our study underscored the integral association between peripheral blood immune cells and the three common UCs at both the genetic and genomic levels. Although clear mechanisms cannot be elucidated through our study, it offers valuable insights for identifying the systemic immunological context of cancer susceptibility and may contribute to the development of blood-based immunological biomarkers and therapeutic targets for future translational studies.

## Figures and Tables

**Table 1:** Heterogeneity and horizontal pleiotropy of positive MR analysis results with immune cells as exposure and UCs as outcome.

Outcomes	Traits	Heterogeneity		Pleiotropy		Outcomes	Traits	Heterogeneity		Pleiotropy	
		I^2^	*p*-Value	Egger Intercept	*p*-Value			I^2^	*p*-Value	Egger Intercept	*p*-Value
Bladder cancer of FinnGen	FSC-A on granulocyte	0	0.946	0.001	0.965	Prostate cancer of FinnGen	CD3 on HLA DR+ CD4+ T cells	0.399	0.005	0.004	0.667
	HLA DR+ CD8br AC	0	0.638	<0.001	0.963		CD19 on IgD- CD38- B cells	0.076	0.355	0.006	0.288
	CD40 on CD14- CD16 + monocyte	0	0.516	0.012	0.161		CD25 on IgD+ CD38- B cells	0.275	0.058	0.005	0.302
	CD39 on CD39 + secreting Treg	0.087	0.188	0.001	0.870		CD127 on CD28- CD8+ T cells	0	0.950	0.000	0.942
	CD39+ resting Treg %resting Treg	0.048	0.310	0.004	0.561		CD40 on CD14+ CD16+ monocytes	0	0.767	−0.003	0.407
	HLA DR on HLA DR+ T cell	0	0.473	−0.013	0.354		IgD on IgD+ CD24- B cells	0.055	0.353	−0.002	0.651
	HVEM on CD45RA- CD4+	0.139	0.271	0.008	0.609		CD33 on CD33dim HLA DR+ CD11b-	0.099	0.219	0.001	0.785
	CD39+ CD4+ %CD4+	0	0.579	0.006	0.371		CD45 on natural killer cells	0.285	0.067	0.003	0.689
	CD27 on sw mem	0.203	0.053	0.013	0.377		CD62L- plasmacytoid dendritic cell absolute count	0	0.891	−0.002	0.753
	HLA DR on CD33br HLA DR+ CD14dim	0.412	<0.001	−0.006	0.731		Plasma blast-plasma cell %B cell	0.024	0.428	0.008	0.249
	CD8dim NKT %T cell	0.092	0.291	−0.008	0.641		CD4+ CD8dim T cell %lymphocyte	0.342	0.007	0.022	0.007
	HLA DR on CD33dim HLA DR+ CD11b+	0.334	0.002	<0.001	0.986		T/B cell	0	0.841	−0.007	0.224
	CD28 on CD28+ CD4+	0	0.835	0.007	0.423		IgD+ CD38dim B cell %B cell	0.366	0.077	−0.004	0.644
	CD27 on unsw mem	0.209	0.059	0.023	0.083		CD28- CD8dim T cell %T cell	0.200	0.150	−0.002	0.745
	CD28 on CD39+ secreting Treg	0.048	0.382	0.002	0.892		CD25 on CD28+ CD4+ T cell	0.184	0.195	−0.002	0.870
	HLA DR on CD33dim HLA DR+ CD11b-	0.198	0.065	−0.008	0.607		Lymphocyte absolute count	0.321	0.089	0.004	0.645
	TD CD8br %CD8br	0.000	0.476	0.016	0.153		CD27 on CD20- B cell	0	0.806	0.010	0.165
	HLA DR on CD14+ monocyte	0.378	<0.001	−0.010	0.450		CX3CR1 on CD14+ CD16- monocyte	0.079	0.308	0.009	0.131
	IgD- CD38 dim %lymphocyte	0	0.738	−0.010	0.391	Prostate cancer of IEU	HLA DR on plasmacytoid dendritic cell	0	0.672	−0.012	0.126
	Lymphocyte AC	0	0.471	−0.003	0.867		HLA DR on dendritic cell	0	0.563	−0.014	0.127
	HVEM on CM CD8br	0.221	0.187	0.003	0.873		HLA DR++ monocyte absolute count	0	0.475	NA	NA
Bladder cancer of IEU	HLA DR+ T cell%T cell	0	0.609	<−0.001	0.430		HLA DR on myeloid dendritic cell	0.329	0.177	−0.028	0.033
	HLA DR+ T cell absolute count	0.062	0.371	<−0.001	0.295		HLA DR on CD33+ HLA DR+ CD14dim	0	0.985	0.003	0.946
	HLA DR+ CD4+ T cell %lymphocyte	0	0.614	NA	NA		HLA DR on CD33+ HLA DR+ CD14-	0	0.984	0.004	0.905
	HLA DR+ CD4+ T cell absolute count	0	0.836	NA	NA		TCRgd T cell absolute count	0	0.757	NA	NA
Kidney cancer of FinnGen	CD45RA- CD4+ T cell %CD4+ T cell	0	0.752	0.002	0.847		HLA DR on monocyte	0	0.660	−0.014	0.432
	Resting CD4 regulatory T cell absolute count	0	0.760	−0.017	0.120		CD4 on CD4+ T cell	0	0.410	NA	NA
	CD28+ CD45RA+ CD8dim T cell %CD8dim T cell	0.137	0.218	−0.007	0.449		FSC-A on natural killer cells	0	0.851	0.001	0.968
	CD3 on terminally differentiated CD8+ T	0.128	0.261	−0.038	0.053		Lymphocyte absolute count	0	0.604	NA	NA
	CD11c on CD62L+ myeloid dendritic cell	0.175	0.158	−0.001	0.947		HLA DR on CD14- CD16-	0.713	0.008	−0.009	0.740
	CD20 on IgD- CD38+ B cell	0	0.731	0.002	0.876		CD64 on CD14+ CD16+ monocytes	0	0.811	NA	NA
	CD62L on CD62L+ Dendritic Cell	0	0.472	−0.027	0.093		T cell absolute count	0	0.549	NA	NA
	Activated and resting CD4 regulatory T cell %CD4+ T cell	0.201	0.113	−0.016	0.161		CD11c on CD62L+ myeloid dendritic cells	0	0.976	NA	NA
	CD39 on CD39+ CD8+ T cell	0.049	0.374	0.030	0.043		HLA DR on CD33dim HLA DR+ CD11b-	0.656	0.020	−0.045	0.203
	CD4+ CD8dim T cell %leukocyte	0.147	0.193	0.024	0.222		HVEM on/naïve CD8+ T cell	0	0.621	NA	NA
	CD24 on IgD+ CD24+ B cell	0.213	0.125	0.022	0.026		Naïve CD8+ T cell %T cell	0	0.901	−0.003	0.771
	CD45RA on CD39+ resting CD4 regulatory T cell	0.110	0.297	−0.024	0.166		HLA DR on CD33dim HLA DR+ CD11b+	0.528	0.096	<0.001	0.999
	Naïve CD4+ T cell %CD4+ T cell	0.034	0.404	−0.014	0.182		HLA DR++ monocyte %monocyte	0	0.455	−0.032	0.431
	CD11c on myeloid dendritic cell	0.292	0.031	0.015	0.171		HLA DR+ natural killer %natural killer	0	0.464	0.006	0.403
	CD45 on HLA DR+ /natural killer	0	0.842	−0.023	0.300		CD14- CD16- AC	0	0.745	−0.062	0.584
	IgD+ CD38- B cell %lymphocyte	0.145	0.264	−0.012	0.425		IgD+ CD38- B cell %B cell	0	0.384	NA	NA
	CD25 on CD39+ activated CD4 regulatory T cell	0.101	0.342	−0.004	0.815						
	CD4 on terminally differentiated CD4+ T cell	0	0.503	−0.008	0.600						
	IgD+ CD24- B cell %B cell	0	0.844	−0.014	0.394						
	CD14-CD16+ monocyte %monocyte	0.157	0.211	0.002	0.880						
	Secreting CD4 regulatory T cell %CD4+ T cell	0.060	0.355	−0.007	0.436						
	HVEM on CD4+ T cell	0.033	0.417	−0.003	0.856						
	Activated and secreting CD4 regulatory T cell %CD4+ T cell	0	0.513	0.009	0.342						

NA = Not available due to insufficient number of SNPs (n < 3) for Egger intercept calculation.

**Table 2:** Result of the reverse MR analysis carried out on immunophenotypes and UC with direct causality.

Trait	nSNP	Beta	SE	*p*-Value	OR (95% CI)
Bladder cancer					
HLA DR+ T cell absolute count	2	−0.016	0.129	0.902	0.984 (0.764–1.268)
HLA DR+ T cell%T cell	2	0.021	0.129	0.873	1.021 (0.792–1.316)
HLA DR+ CD4+ T cell absolute count	2	0.012	0.196	0.950	1.012 (0.690–1.486)
HLA DR+ CD4+ T cell %lymphocyte	2	0.015	0.177	0.930	1.016 (0.718–1.438)
Prostate cancer					
Plasma blast-plasma cell %B cell	54	0.053	0.039	0.178	1.054 (0.976–1.138)
CD62L- plasmacytoid dendritic cell absolute count	54	0.007	0.029	0.799	1.007 (0.952–1.066)
Naive CD8+ T cell %T cell	55	0.016	0.021	0.436	1.016 (0.976–1.058)
T/B cell	55	−0.003	0.031	0.920	0.997 (0.938–1.059)
Lymphocyte absolute count	55	−0.076	0.029	0.010	0.927 (0.875–0.981)
T cell absolute count	55	−0.058	0.029	0.045	0.944 (0.892–0.999)
CD19 on IgD- CD38- B cell	54	0.026	0.029	0.384	1.026 (0.969–1.086)
CD25 on IgD+ CD38- B cell	54	−0.016	0.032	0.613	0.984 (0.924–1.048)
IgD on IgD+ CD24- B cell	54	0.034	0.030	0.255	1.035 (0.976–1.097)
CD3 on HLA DR+ CD4+ T cell	54	−0.017	0.032	0.606	0.983 (0.923–1.048)
CD45 on natural killer	54	−0.029	0.032	0.361	0.971 (0.912–1.034)
CD40 on CD14+ CD16+ monocyte	54	−0.035	0.030	0.240	0.966 (0.911–1.023)
CX3CR1 on CD14+ CD16- monocyte	54	−0.083	0.030	0.006	0.920 (0.868–0.976)
CD64 on CD14+ CD16+ monocyte	52	0.001	0.030	0.985	1.001 (0.944–1.060)

Note: SE, Standard error.

## Data Availability

Data on immune cells were sourced from the publicly accessible GWAS Catalog (https://www.ebi.ac.uk/gwas/ (accessed on 16 February 2025)), covering registration numbers from GCST90001391 to GCST90002121. The genetic data regarding three common urological cancers were sourced from the FinnGen website (https://r10.finngen.fi/ (accessed on 16 February 2025)) and the Open GWAS database (https://gwas.mrcieu.ac.uk/ (accessed on 16 February 2025)). The GWAS IDs for the Open GWAS database are ieu-b-4874, ieu-b-85, and ukb-b-1316.

## Supplementary Materials

The following supporting information can be downloaded at https://www.mdpi.com/article/10.3390/biomedicines13061480/s1.
