# Supplementary material for: Immune System Alterations in the Development of Three Urological Cancers: Insights from Large-Sample Mendelian Randomization
Source: Biomedicines. 2025 Jun 16;13(6):1480. doi: 10.3390/biomedicines13061480 (PMC12191397; doi:10.3390/biomedicines13061480)
Supplement: Supplementary file 1 [file biomedicines-13-01480-s001.zip › Supplementary Table 25.pdf]

Supplementary Table 25. Significant MR results for PC in the BBJ cohort.

Trait	nSNP	Beta	SE	*p*-value	OR (95% CI)
CD28+ CD45RA- CD8+ T cell Absolute Count	13	0.126	0.040	0.002	1.134 (1.047-1.227)
PDL-1 on CD14- CD16-	9	-0.159	0.055	0.004	0.853 (0.766-0.951)
Resting CD4 regulatory T cell %CD4 regulatory T cell	22	-0.075	0.027	0.005	0.927 (0.880-0.977)
Terminally Differentiated CD4-CD8- T cell %CD4-CD8- T cell	14	-0.114	0.044	0.010	0.892 (0.818-0.973)
SSC-A on CD14+ monocyte	33	-0.082	0.031	0.010	0.922 (0.867-0.980)
CD45 on CD8+ T cell	7	0.118	0.047	0.011	1.125 (1.027-1.233)
CD16-CD56 on Natural Killer T	18	0.079	0.032	0.013	1.082 (1.01-1.152)
CD45 on CD33dim HLA DR-	8	-0.187	0.076	0.015	0.830 (0.714-0.96)
CD86 on granulocyte	11	-0.110	0.047	0.018	0.895 (0.817-0.981)
CCR7 on naive CD4+ T cell	11	-0.073	0.031	0.019	0.930(0.875-0.988)
CD25 on CD39+ resting CD4 regulatory T cell	5	0.226	0.098	0.021	1.253 (1.03-1.519)
CD127- CD8+ T cell Absolute Count	29	0.068	0.030	0.022	1.070 (1.010-1.134)
CD80 on myeloid Dendritic Cell	18	0.062	0.027	0.022	1.064 (1.009-1.122)
CD45 on B cell	21	-0.073	0.032	0.022	0.930 (0.873-0.990)
CD28 on CD45RA+ CD4+ T cell	10	-0.090	0.039	0.023	0.914 (0.846-0.987)
CX3CR1 on CD14+ CD16+ monocyte	24	0.033	0.015	0.023	1.034 (1.005-1.064)
SSC-A on HLA DR+ CD8+ T cell	12	-0.106	0.047	0.026	0.900 (0.820-0.987)
CD80 on granulocyte	12	-0.078	0.036	0.029	0.925 (0.862-0.992)
CD28- CD127- CD25++ CD8+ T cell %T cell	8	-0.152	0.070	0.031	0.859 (0.748-0.986)
Granulocyte %leukocyte	12	0.072	0.034	0.032	1.075 (1.006-1.148)
FSC-A on B cell	13	0.098	0.046	0.033	1.103 (1.008-1.206)
Natural Killer Absolute Count	10	0.102	0.049	0.036	1.108 (1.007-1.219)
Secreting CD4 regulatory T cell %CD4+ T cell	19	-0.012	0.009	0.037	0.988 (0.977-0.999)
Unswitched memory B cell %lymphocyte	16	0.081	0.0399	0.038	1.084 (1.005-1.171)
CD45 on HLA DR+ CD4+ T cell	14	0.091	0.044	0.039	1.095 (1.005-1.193)
FSC-A on granulocyte	14	-0.064	0.031	0.039	0.938 (0.882-0.997)
CD3 on naive CD8+ T cell	38	-0.043	0.021	0.042	0.958 (0.919-0.998)
Activated & secreting CD4 regulatory T cell %CD4+ T cell	19	-0.012	0.006	0.047	0.988 (0.977-1.000)
HLA DR on CD14+ CD16- monocyte	90	-0.027	0.014	0.047	0.973 (0.947-1.000)
Natural Killer T Absolute Count	19	-0.044	0.022	0.049	0.957 (0.916-1.000)
